# Supplementary material for: Improving training on hepatitis B research in Nigeria: Findings from an innovation bootcamp to strengthen capacity
Source: PLOS Glob Public Health. 2025 Nov 25;5(11):e0004883. doi: 10.1371/journal.pgph.0004883 (PMC12646463; doi:10.1371/journal.pgph.0004883)
Supplement: S1 Table — (DOCX) [file pgph.0004883.s002.docx]

**S1 Table: Summary of pre- and post-surveys assessing skills of bootcamp participants**

***Please rank how skilled you feel in Research for each of the competencies below.***

| **Variable** | **Pre (N=15)** | **Post (N=15)** |
| --- | --- | --- |
| **Research methods in the health sciences** |  |  |
| Moderately skilled | 9 (60.0%) | 6 (40.0%) |
| Not at all skilled | 0 (0%) | 0 (0%) |
| Skilled | 4 (26.7%) | 6 (40.0%) |
| Somewhat skilled | 2 (13.3%) | 2 (13.3%) |
| Very skilled | 0 (0%) | 1 (6.7%) |
| **Conducting literature reviews** |  |  |
| Moderately skilled | 4 (26.7%) | 5 (33.3%) |
| Not at all skilled | 2 (13.3%) | 0 (0%) |
| Skilled | 4 (26.7%) | 6 (40.0%) |
| Somewhat skilled | 4 (26.7%) | 2 (13.3%) |
| Very skilled | 1 (6.7%) | 2 (13.3%) |
| **Completing SWOT analyses from community data** |  |  |
| Moderately skilled | 3 (20.0%) | 4 (26.7%) |
| Not at all skilled | 7 (46.7%) | 0 (0%) |
| Skilled | 1 (6.7%) | 7 (46.7%) |
| Somewhat skilled | 4 (26.7%) | 1 (6.7%) |
| Very skilled | 0 (0%) | 3 (20.0%) |
| **Completing an ethical approval proposal** |  |  |
| Moderately skilled | 4 (26.7%) | 3 (20.0%) |
| Not at all skilled | 3 (20.0%) | 2 (13.3%) |
| Skilled | 1 (6.7%) | 4 (26.7%) |
| Somewhat skilled | 7 (46.7%) | 5 (33.3%) |
| Very skilled | 0 (0%) | 1 (6.7%) |
| **Distinguishing between different qualitative research methods** |  |  |
| Moderately skilled | 5 (33.3%) | 5 (33.3%) |
| Not at all skilled | 2 (13.3%) | 0 (0%) |
| Skilled | 2 (13.3%) | 7 (46.7%) |
| Somewhat skilled | 6 (40.0%) | 1 (6.7%) |
| Very skilled | 0 (0%) | 2 (13.3%) |
| **Conducting qualitative data analysis** |  |  |
| Moderately skilled | 5 (33.3%) | 4 (26.7%) |
| Not at all skilled | 5 (33.3%) | 2 (13.3%) |
| Skilled | 2 (13.3%) | 8 (53.3%) |
| Somewhat skilled | 3 (20.0%) | 0 (0%) |
| Very skilled | 0 (0%) | 1 (6.7%) |
| **Conducting quantitative data analysis** |  |  |
| Moderately skilled | 2 (13.3%) | 7 (46.7%) |
| Not at all skilled | 5 (33.3%) | 0 (0%) |
| Skilled | 4 (26.7%) | 6 (40.0%) |
| Somewhat skilled | 4 (26.7%) | 0 (0%) |
| Very skilled | 0 (0%) | 2 (13.3%) |
| **Using quantitative data analysis software** |  |  |
| Moderately skilled | 5 (33.3%) | 1 (6.7%) |
| Not at all skilled | 6 (40.0%) | 6 (40.0%) |
| Skilled | 1 (6.7%) | 1 (6.7%) |
| Somewhat skilled | 3 (20.0%) | 5 (33.3%) |
| Very skilled | 0 (0%) | 2 (13.3%) |
| **Producing quantitative data visualizations** |  |  |
| Moderately skilled | 4 (26.7%) | 5 (33.3%) |
| Not at all skilled | 7 (46.7%) | 2 (13.3%) |
| Skilled | 1 (6.7%) | 2 (13.3%) |
| Somewhat skilled | 3 (20.0%) | 3 (20.0%) |
| Very skilled | 0 (0%) | 3 (20.0%) |
| **Selecting human-centered design methods for a specific problem or community** |  |  |
| Moderately skilled | 3 (20.0%) | 8 (53.3%) |
| Not at all skilled | 2 (13.3%) | 0 (0%) |
| Skilled | 3 (20.0%) | 3 (20.0%) |
| Somewhat skilled | 7 (46.7%) | 3 (20.0%) |
| Very skilled | 0 (0%) | 1 (6.7%) |
| **Conducting human-centered design activities** |  |  |
| Moderately skilled | 5 (33.3%) | 6 (40.0%) |
| Not at all skilled | 2 (13.3%) | 0 (0%) |
| Skilled | 2 (13.3%) | 6 (40.0%) |
| Somewhat skilled | 6 (40.0%) | 1 (6.7%) |
| Very skilled | 0 (0%) | 2 (13.3%) |
| **Engaging stakeholders in research** |  |  |
| Moderately skilled | 5 (33.3%) | 3 (20.0%) |
| Not at all skilled | 3 (20.0%) | 0 (0%) |
| Skilled | 1 (6.7%) | 7 (46.7%) |
| Somewhat skilled | 6 (40.0%) | 1 (6.7%) |
| Very skilled | 0 (0%) | 4 (26.7%) |
| **Utilizing storytelling for health promotion** |  |  |
| Moderately skilled | 5 (33.3%) | 3 (20.0%) |
| Not at all skilled | 0 (0%) | 0 (0%) |
| Skilled | 3 (20.0%) | 7 (46.7%) |
| Somewhat skilled | 7 (46.7%) | 2 (13.3%) |
| Very skilled | 0 (0%) | 3 (20.0%) |
| **Writing a research proposal** |  |  |
| Moderately skilled | 5 (33.3%) | 3 (20.0%) |
| Not at all skilled | 2 (13.3%) | 3 (20.0%) |
| Skilled | 0 (0%) | 6 (40.0%) |
| Somewhat skilled | 8 (53.3%) | 3 (20.0%) |
| Very skilled | 0 (0%) | 0 (0%) |
| **Budgeting for a research project or intervention** |  |  |
| Moderately skilled | 2 (13.3%) | 4 (26.7%) |
| Not at all skilled | 4 (26.7%) | 1 (6.7%) |
| Skilled | 0 (0%) | 4 (26.7%) |
| Somewhat skilled | 9 (60.0%) | 3 (20.0%) |
| Very skilled | 0 (0%) | 3 (20.0%) |

***Please rank how knowledgeable you feel about* HBV-related Knowledge**

***in each of the following competencies***

| **Variable** | **Pre (N=15)** | **Post (N=15)** |
| --- | --- | --- |
| **Basics of HBV infection and epidemiology among newborns in Nigeria** |  |  |
| Moderately knowledgeable | 5 (33.3%) | 2 (13.3%) |
| Slightly knowledgeable | 1 (6.7%) | 1 (6.7%) |
| Very knowledgeable | 9 (60.0%) | 8 (53.3%) |
| Extremely knowledgeable | 0 (0%) | 4 (26.7%) |
| **The health effects of HBV infection on newborns and adults in Nigeria** |  |  |
| Extremely knowledgeable | 1 (6.7%) | 4 (26.7%) |
| Moderately knowledgeable | 5 (33.3%) | 2 (13.3%) |
| Slightly knowledgeable | 1 (6.7%) | 0 (0%) |
| Very knowledgeable | 8 (53.3%) | 8 (53.3%) |
| Not at all knowledgeable | 0 (0%) | 1 (6.7%) |
| **Challenges and barriers to HBV vaccination in newborns in Nigeria** |  |  |
| Moderately knowledgeable | 2 (13.3%) | 3 (20.0%) |
| Slightly knowledgeable | 2 (13.3%) | 0 (0%) |
| Very knowledgeable | 11 (73.3%) | 7 (46.7%) |
| Extremely knowledgeable | 0 (0%) | 5 (33.3%) |
| **Misconceptions and myths related to HBV vaccination of newborns in Nigeria** |  |  |
| Extremely knowledgeable | 1 (6.7%) | 4 (26.7%) |
| Moderately knowledgeable | 3 (20.0%) | 1 (6.7%) |
| Not at all knowledgeable | 1 (6.7%) | 0 (0%) |
| Slightly knowledgeable | 1 (6.7%) | 1 (6.7%) |
| Very knowledgeable | 9 (60.0%) | 9 (60.0%) |
| **Facilitators (drivers) of HBV vaccinations among newborns in Nigeria** |  |  |
| Extremely knowledgeable | 2 (13.3%) | 4 (26.7%) |
| Moderately knowledgeable | 4 (26.7%) | 4 (26.7%) |
| Not at all knowledgeable | 1 (6.7%) | 0 (0%) |
| Slightly knowledgeable | 2 (13.3%) | 0 (0%) |
| Very knowledgeable | 6 (40.0%) | 7 (46.7%) |
| **Existing interventions to promote HBV vaccinations among newborns in Nigeria** |  |  |
| Extremely knowledgeable | 1 (6.7%) | 2 (13.3%) |
| Moderately knowledgeable | 7 (46.7%) | 6 (40.0%) |
| Not at all knowledgeable | 1 (6.7%) | 1 (6.7%) |
| Slightly knowledgeable | 1 (6.7%) | 0 (0%) |
| Very knowledgeable | 5 (33.3%) | 6 (40.0%) |
| **Key communities or community members that can help drive HBV vaccinations in newborns in Nigeria** |  |  |
| Moderately knowledgeable | 5 (33.3%) | 5 (33.3%) |
| Not at all knowledgeable | 2 (13.3%) | 0 (0%) |
| Slightly knowledgeable | 3 (20.0%) | 0 (0%) |
| Very knowledgeable | 5 (33.3%) | 4 (26.7%) |
| Extremely knowledgeable | 0 (0%) | 6 (40.0%) |
| **The role of mothers in HBV vaccinations among newborns in Nigeria** |  |  |
| Extremely knowledgeable | 1 (6.7%) | 7 (46.7%) |
| Moderately knowledgeable | 3 (20.0%) | 0 (0%) |
| Slightly knowledgeable | 1 (6.7%) | 0 (0%) |
| Very knowledgeable | 10 (66.7%) | 8 (53.3%) |
| **The role of fathers in HBV vaccinations among newborns in Nigeria** |  |  |
| Extremely knowledgeable | 1 (6.7%) | 5 (33.3%) |
| Moderately knowledgeable | 5 (33.3%) | 2 (13.3%) |
| Slightly knowledgeable | 2 (13.3%) | 0 (0%) |
| Very knowledgeable | 7 (46.7%) | 8 (53.3%) |
| **The role of other family members in HBV vaccinations among newborns in Nigeria** |  |  |
| Moderately knowledgeable | 6 (40.0%) | 1 (6.7%) |
| Not at all knowledgeable | 1 (6.7%) | 0 (0%) |
| Slightly knowledgeable | 1 (6.7%) | 0 (0%) |
| Very knowledgeable | 7 (46.7%) | 10 (66.7%) |
| Extremely knowledgeable | 0 (0%) | 4 (26.7%) |
| **Program components that can drive the sustainability of HBV vaccination interventions for newborns in Nigeria** |  |  |
| Moderately knowledgeable | 7 (46.7%) | 2 (13.3%) |
| Not at all knowledgeable | 1 (6.7%) | 0 (0%) |
| Slightly knowledgeable | 3 (20.0%) | 1 (6.7%) |
| Very knowledgeable | 4 (26.7%) | 7 (46.7%) |
| Extremely knowledgeable | 0 (0%) | 5 (33.3%) |
