# Supplementary material for: Improving training on hepatitis B research in Nigeria: Findings from an innovation bootcamp to strengthen capacity
Source: PLOS Glob Public Health. 2025 Nov 25;5(11):e0004883. doi: 10.1371/journal.pgph.0004883 (PMC12646463; doi:10.1371/journal.pgph.0004883)
Supplement: S2 Table — (DOCX) [file pgph.0004883.s003.docx]

**S2 Table: Implementation Strategies proposed by four teams in relation to the PEN-3 Model**

|  | **Team** | **Location** | **Strategies Proposed** |
| --- | --- | --- | --- |
| 1. | **HEPCRUSADER** | Ovoko community, Nsukka | - Work with healthcare professionals and traditional birth attendants to facilitate collaborations, educate community gatekeepers, and aid with vaccination processes. (PEN-3 Enabler) - Train and empower a network of Community Gatekeepers as ambassadors to effectively enlighten community members about the HBV-BD vaccine (PEN-3 Perceptions) - Conduct workshops in health centers and communities for health workers, pregnant women, and family members, emphasizing the importance of HB-BD vaccination, its role in preventing liver cancer, and addressing any concerns or misconceptions. (PEN-3 Enabler & Nurturer) - Develop and distribute educational materials such as flyers and posters to enhance knowledge and dispel myths. (PEN-3 Perceptions) - Address common misconceptions about HBV and the HB-BD vaccine and ensure the public is well informed. (PEN-3 Perceptions) |
| 2. | **HEROMOMS** | Ile Ife, Osun State | **Training Workshops for TBAs:**   - TBAs will be trained on HBV transmission, prevention, and the importance of timely HBV-BD vaccination. (PEN-3 Enabler) - Emphasis on the role of TBAs in referring newborns to health facilities for vaccination. (PEN-3 Enabler) - Hands-on demonstrations on counseling pregnant women and new mothers. (PEN-3 Enabler) - Develop and distribute educational materials such as flyers and posters to enhance knowledge and dispel myths. (PEN-3 Perceptions)   **Integration into Health Facilities:**   - TBAs will be linked with nearby health facilities. (PEN-3 Enabler) - Establish a structured referral system where TBAs track and refer newborns for HBV-BD vaccination. (PEN-3 Enabler) - Health workers will coordinate with TBAs to ensure vaccination uptake. (PEN-3 Enabler) |
| 3. | **HEXA** | Ile Ife, Osun State | **Community Engagement and Education**.   - Organize community dialogues, town hall meetings, and sensitization programs involving mothers, caregivers, and key stakeholders. (PEN-3 Nurturer) - Develop and distribute educational materials such as flyers and posters to enhance knowledge and dispel myths. (PEN-3 Perceptions)   **Referral System Linking Traditional Birth Attendants (TBAs) to Primary Healthcare**   - Establish a structured referral pathway where TBAs direct newborns to nearby primary healthcare centers (PHCs) for timely HBV-BD vaccination. (PEN-3 Enabler) - Provide TBAs with referral cards and tracking tools to monitor the vaccination status of newborns. (PEN-3 Enabler) - Ensure collaboration between TBAs and healthcare workers to improve vaccination follow-ups. (PEN-3 Enabler)   **Capacity Building for Traditional Birth Attendants (TBAs)**   - Organize training workshops for TBAs on HBV transmission, prevention, and newborn vaccination. (PEN-3 Enabler) - Equip TBAs with the knowledge to educate pregnant women and caregivers about HBV and vaccine uptake. (PEN-3 Nurturer)   **Partnership with Medical Women’s Association of Nigeria (MWAN), Osun State Chapter**   - Leverage the expertise of MWAN to develop and conduct structured training programs for TBAs. (PEN-3 Enabler) - MWAN will facilitate evidence-based capacity-building sessions to strengthen TBAs' knowledge and referral practices. (PEN-3 Enabler) - Collaborate with MWAN to advocate for policy support and increased funding for TBA integration in vaccination programs. (PEN-3 Enabler) |
| 4. | **MCHILD** | Ona Ara, Oyo State | **Awareness and Engagement:**   - Conduct health talks in local maternity homes for pregnant women and caregivers accessing care through this facility. (PEN-3 Nurturer) - Community engagements during social events to introduce the study to caregivers, focusing on HBV, the importance of HBV vaccination, its role in preventing liver cancer, reinforcing the slogan “One life, One liver” and addressing any concerns or misconceptions. (PEN-3 Perceptions)   **Community-Based Vaccination:**   - Collaborating with local immunization officers to facilitate timely HBV vaccination within 24 hours of birth at the Local maternity homes, while adhering to the recommended HBV vaccine schedule in a partnering health facility (Ona-ara local primary health centers). (PEN-3 Enabler)   **Follow-Ups:**   - Traditional Birth attendants and Ayanfe (an innovative chatbot) through timely reminders to mothers/ Caregivers will follow up ensuring adherence and completion of vaccination schedules at important intervals. (PEN-3 Nurturer) |
