# Supplementary material for: Improving training on hepatitis B research in Nigeria: Findings from an innovation bootcamp to strengthen capacity
Source: PLOS Glob Public Health. 2025 Nov 25;5(11):e0004883. doi: 10.1371/journal.pgph.0004883 (PMC12646463; doi:10.1371/journal.pgph.0004883)
Supplement: Attachment — Submitted filename: PGPH-D-25-01335_reviewer_comments.pdf [file pgph.0004883.s004.pdf]

Reviewer comments to Author:

Thank you for the opportunity to review this study. Overall, this manuscript described an innovative approach to engaging the community, specifically the youth, to generate creative solutions to addressing barriers to hepatitis B birth dose vaccine uptake among newborns. The approach was a 5-week bootcamp that comprised of activities aimed at building research capacity among young people and supporting the implementation of developed strategies. Though this approach was unique, it is unclear if the authors achieved the study aim, specifically if youth implemented develop strategies as this is not described in the manuscript. Please find below a detailed list of suggested revisions to help strengthen this manuscript.

The following are suggested revisions:

Title:

- The title does not accurately describe what is discussed throughout the manuscript. The title references improved training on hepatitis B research, but the manuscript is focused on the implementation of a bootcamp. Consider revising the title to better match the contents of the manuscript.

Abstract:

- Abstract is 320 words; it must not exceed 300. Please refer to submission guidelines here: https://journals.plos.org/globalpublichealth/s/submission-guidelines and revise the abstract to 300 words or less.
- Line 61: The abstract mentions a hepatitis B birth dose vaccination coverage of 30% in Nigeria, which is incorrect. According to the WUENIC data, Hepatitis B birth dose vaccination coverage within 24 hours of birth in Nigeria was 52% in 2023 and has been consistent since 2019. The reference included in the manuscript also shows vaccination coverage at 52% from 2019 – 2021. Please review the included citation (#7) as well as WHO WUENIC data on Hepatitis B birth dose vaccination coverage in Nigeria and correct this statement.
- Line 78-81: conclusion should speak directly to study aim. If implementation of developed strategies were not complete in time for this manuscript then the study aim should be revised to speak to what this manuscript is describing.

Introduction:

- Line 104: include year of HBV prevalence referenced here.
- Line 107-109: “HBV vaccine initiated within 24 hours of birth, followed by three additional doses (typically at 6, 10, and 14 weeks after birth), provides durable immunity, substantially decreasing the risk of HBV and the incidence of liver cancer in nearly 100% of cases [5].” The proportion of ‘nearly 100% of cases’ does not seem accurate and is not seen in the included citation. Would suggest reviewing the 2017

WHO position paper on Hepatitis B vaccines and possibly referencing that document instead of the reference from HHS. Please confirm proportion and correct based on updated reference.

- Line 109-111: Suggest including timeframe for this statement since the number of countries with HepB-BD in their immunization schedule has increased from 14 and will continue to increase given Gavi’s support of HepB-BD introduction.
- Line 113-114: see comment in abstract section about this statistic. It is currently incorrect and needs to be revised.
- Line 114-115: “The failure to engage end-users in planning and implementation is a major driver of unsuccessful interventions addressing low vaccination coverage [5].” In the case of HepB-BD, aren’t the end-users newborns? Not sure if this statement makes sense in this context, consider revising.
- Line 116: Not familiar with terms “Global South” and Global North”, please define what countries are being referred to here.
- Line 118-119: “Many projects in the global South have failed or faced problems because the primary beneficiaries were not involved in initiating, designing, and delivering the project [8]”. Again, in the case of HepB-BD, the primary beneficiaries (e.g., newborns) aren’t able to be involved in initiating, designing, or delivering the project. This statement does not make sense in this context, consider revising or removing.
- Line 126: “young people represent a significant portion of the population; 45.46% are between 15 and 35 years old”. Please change ‘are’ to ‘were’ since referencing a proportion from 2021.
- The connection between young people and how they can engage the community or drive community change needs to be emphasized. Line 121-122 speaks to community ownership overall but the connection with youth needs to be strengthen in the introduction to rationalize why they are the best target audience for this study.
- Line 145-146: what is hepatitis B research training? This term is vague and does not clearly convey what is being advanced. Is it training others on how to do hepatitis B research or training others on hepatitis B burden and disease? Please clarify term.
- Line 146: “By outlining our journey from generating ideas to conducting pilot fieldwork…” the outline for conducting pilot fieldwork is missing in this manuscript. Since the bootcamp was conducted a year ago (June-July 2024) perhaps there is more substantial data available from pilots that can be included in this manuscript write up. Please include an outline and description of pilot projects for context.

Materials and Methods:

- Line 152: Was the goal focused on overall HepB-BD vaccination or timely (within 24 hours)?
- It is unclear if there was any specific training/education/sensitization included in the bootcamp to educate youth on hepatitis B burden, disease, and prevention. This detail needs to be explained in the methods section. Were youth expected to know anything about hepatitis B before being tasked with generating innovative ways to improve HepB-BD uptake?
- Line 166-169: Run-on sentence. Please rephrase for clarity.
- Line 172-173: "The research team received 349/396 submissions electronically (WhatsApp, email, and the website) and 11/396 in person". The numbers 349 + 11 add up to 360 not 396. Where did the other 36 entries come from? Also, what is meant by "the website"?
- Line 174-175: Include a description of how all entries were scanned for AI usage. What was used for this scan? Include citation of tool, if available.
- Line 177 implies that all entries were submitted by a "team". Was this part of the submission requirements? Were youth asked to submit as a team vs an individual? This needs to be better explained in the methods.
- Line 179-181 implies that finalists were tasked with designing and refining their strategies prior to the bootcamp, where all the research training took place. Were they provided feedback on their idea or given any guidance during the Designathon? The order of operations for this study is unclear, please clarify.
- Line 180-181: finalists were asked to refine strategies to enhance HepB-BD uptake and awareness within Nigerian communities. Were communities specified in any way or decided upon based on team? Could imagine that communities across Nigeria vary greatly and would require different approaches. Since teams were all from Southern Nigeria, did their interventions only focus on these communities? Southern Nigeria has higher rates of HepB-BD vaccination uptake compared to Northern Nigeria (according to 2021 MICS/NICS report), this is an important point that should be mentioned in the discussion of the paper, perhaps as a limitation given the lack of attention provided to communities in Northern Nigeria, where the burden of hepatitis B is greater.
- Line 191: "The five teams that made it past the Designathon…" this phrase needs to be better explained. Did the other 5 teams drop out? Were they lost to follow up?
- Line 196-198: Were team facilitators knowledgeable about hepatitis B at all? Were they provided any training or education on the disease burden or overall prevention strategy? If so, this needs to be explained in the methods section.

- Line 232: “The judges comprised community leaders...”, should read “The judges comprised ‘of’ community leaders”.
- Line 248: It is unclear who the ‘target population’ is. Does it vary by team? Is it mothers, caregivers, newborns, community leaders, traditional birth attendants?
- Line 259: Placement needs to be better explained in this paragraph. Was it that teams were judged and if received first place they were given seed funding to pilot their proposed strategies? How does this fit with the timeline presented in table 1?
- Line 271-272: A virtual refresher course was completed months after the bootcamp ended. Please describe the expected timeline of implementing the pilot. If selected for first place for example, what is the expected timeline for the pilot? Is it after the refresher course is completed?
- Line 291: indicate at what p-value was an estimate considered statistically significant.
- Line 318: the term ‘existential’ should be defined in line 310 where it is first mentioned rather than in line 318.

Results:

- Line 328 mentions that 5 teams completed the bootcamp but in line 261 it mentions that all but one team submitted study proposals that were approved by the research team.
- The study emphasizes the focus on youth; however, the mean age of participants is 25.5 years (ranging from 20 – 53 years old). Given this age, participants were all adults, rather than youths. This is an important clarification that changes the overall assertion of the manuscript and needs to be addressed in the discussion.
- Line 339: Table 2 includes min/max but the text references IQR. Would suggest being consistent on which one is reported. If using IQR, be sure to spell out acronym at first use.
- Line 340: were the difference in pre-test scores described here significant based on the exact test performed? This needs to be stated and p-value included, if yes.
- Table 3: In the post column, there is a maximum research skills score of 101. Is it possible to score over 100? Are these numbers and proportions correct? This needs to be clarified in the table.
- Please be sure to be consistent with the number of places after the decimal point for all proportions presented in Tables.
- Line 351-354: Are these open-ended questions or themes? If not, then %’s should also be included to have a sense of how many participants mentioned the same topic.

- Line 375: Again, how was youth defined in this manuscript? Based on the age of participants presented in Table 2, individuals are technically adults, not youth.
- Table 5: Under the perceptions domain, TBAs are listed under existential but “believe in traditional/alternative medicine” is listed under negative. This seems discordant, please address/clarify in text if this is indeed the correct placement for each.
- Table 5: This table would be easier to read if each new line was denoted with a bullet point

Discussion:

- Line 397: Again, not sure authors can make the claim that this bootcamp was youth led given the ages of the participants. Either define the accepted age range for youth in the introduction of this manuscript or rephrase/remove the term youth as a descriptor for participants.
- Line 404: Again, the notion of ‘involving the audience in design’ is not applicable here as the target audience for HepB-BD uptake is newborns. If the target audience is mothers/caregivers then the argument of involving the audience works –although it would mean that teams were led by reproductive-aged participants rather than youths. Please make sure it is clear through the manuscript who the target audience/primary beneficiaries are.
- Line 442: Is there a reference that can be included for the “Ayanfe” chatbot referenced here? Was this chatbot created by the team for the purpose of this project or did it already exist? This needs to be clearly explained in the manuscript.
- Line 462: Is the chatbox referenced here the same chatbox mentioned in line 442? If not, include name of the chatbox and citation if available.
- The discussion describes what was planned and proposed but does not speak to what was implemented at all. This was part of the study aim and described in the methods but is missing from the results and discussion sections of the paper. Please include.
- Line 473-474: Again, the claim of youth-driven research needs to be modified given the ages of participants. Also, a reference is needed for the statement “Nigeria has a large youth population,” be sure to include the age range for the ‘large youth population’ referenced.
- Line 477: Include citation for Ayange Chatbot if available.
- Line 493: Did the bootcamp address any of the mentioned challenges? Were these challenges faced by implementing teams or just stated from previous literature? Were teams expected to design intervention with common challenges in mind? Please clarify in the text.

- Line 502: The “execution of intervention strategies” were not clearly described in this manuscript. Please include.

Conclusion:

- Line 516-517: This is the one sentence in the manuscript that mentions that piloting of strategies is occurring. This should be described in more detail in the methods and results sections, describing the expected timeline, location of implementation, and any other relevant details.
