# Supplementary material for: Improving training on hepatitis B research in Nigeria: Findings from an innovation bootcamp to strengthen capacity
Source: PLOS Glob Public Health. 2025 Nov 25;5(11):e0004883. doi: 10.1371/journal.pgph.0004883 (PMC12646463; doi:10.1371/journal.pgph.0004883)
Supplement: Attachment — Submitted filename: Rebuttal Letter PLOS.docx [file pgph.0004883.s005.docx]

Response to PLOS on the manuscript “Improving training on hepatitis B research in Nigeria: Findings from an innovation bootcamp to strengthen capacity”

Dear reviewers,

Thank you for the opportunity to revise our manuscript and for thoroughly reviewing the manuscript. The authors sincerely appreciate your hard work, valuable suggestions, constructive comments and corrections, which we have addressed in our responses below. The authors hope that the responses will satisfy all the reviewers’ queries. References to lines for specific changes refer to the line numbering in the clean manuscript version.

**Reviewer # 1:** This is a promising and timely contribution to implementation science, particularly in LMIC contexts. The use of participatory frameworks and youth engagement offers a novel capacity-building model. However, the paper in its current form has serious methodological and reporting limitations that must be addressed to meet the standards of empirical rigor and replicability required for publication in PLOS Global Public Health.

Response: Thank you for recognizing the significance and novelty of the study. The authors are willing to address the concerns about the methodology and limitations of the study

**Title and Abstract
Comment # 1:** The abstract lacks structured headings, which limits clarity. Consider using a structured format (e.g., Background, Methods, Results, Conclusion).

**Response # 1:** We appreciate the reviewer for your comment. The [PLOS Global Public Health submission guideline for abstracts](https://journals.plos.org/globalpublichealth/s/submission-guidelines) states: “While the Abstract is conceptually divided into three sections (Background, Methodology/Principal Findings, and Conclusions/Significance), do not apply these distinct headings to the Abstract within the article file.” For this reason, the abstract was written in plain text.

**Comment # 2:** The study objective is unclear; the abstract shifts between describing the bootcamp and reporting pre-post results. Clarify whether the manuscript primarily evaluates outcomes or describes the intervention design.

**Response # 2:** The study objectives were to describe the activities of the bootcamp in co-designing implementation strategies and to evaluate its impact on increasing research capacity. The abstract has been revised for better clarity and flow. Lines 66-68.

**Comment # 3:** The phrasing of effectiveness is somewhat overstated, given the small sample size and lack of a comparator.

**Response # 3:** We have revised the language to more carefully describe the outcomes as suggestive of potential impact rather than conclusive effectiveness. Our goal is to emphasize promising trends while acknowledging the need for further research with larger samples. Lines 81-85.

**Comment # 4:** Key terms like "participatory action research," "PEN-3 model," and "designathon" are introduced without context or brief explanation, which may confuse non-specialist readers.

**Response # 4:** We thank the reviewer for their comment. The PAR and PEN-3 are described further in the manuscript text. Due to the limited word count, these were not detailed in the abstract. Lines 128-140, 341-365.

**Introduction**
**Comment # 5:** The public health significance of low HepB-BD coverage is clearly articulated with recent data. However, the introduction does not clearly define the research gap. It would help to explicitly state what has not been done before and how this bootcamp fills that gap.

**Response # 5:** Thank you for this valid observation. The gap is that, despite being freely available, the delivery and uptake of the hepatitis B birth dose (HepB-BD) vaccine in Nigeria remain suboptimal, as mentioned in the introduction section. Lines 144-151.

**Comment # 6:** Several frameworks (PAR, PEN-3, Mega-Designathon) are introduced, but their relationships and rationale are not well synthesized. A visual model or conceptual diagram could help orient readers.

**Response # 6:** A visual scheme has been added for clarity. Legend: Conceptual framework integrating cultural constructs of PEN-3 model with participatory research strategies—Crowdsourcing, Designathon (Apprenticeship), and PAR—to co-create, implement, and evaluate interventions aimed at increasing uptake of the hepatitis B birth dose vaccination. Lines 367-370. Refer to Fig4 in supplementary files.

**Comment # 7**: The study objective, while stated, is buried in the final paragraph. Consider rephrasing it as a concise, standalone aim statement.
**Response # 7**: The study aim has been written in a separate paragraph at the end of the introduction, as suggested. Lines 149-154.

**Methods**

**Comment #8:** Study Design: The manuscript claims a "mixed methods design informed by PAR," but the structure lacks coherence. The study more closely resembles a descriptive implementation study with embedded pre-post assessments and thematic analysis. No formal mixed methods integration is presented.
**Response # 8**: The authors have revised the mixed-method statement as the study is essentially a description of the bootcamp activities. Line 66, 149-151.

**Comment # 9:** Sample Selection: Participant recruitment is described in detail, but several concerns arise:
. Selection appears based on submission to an open call, which may introduce self-selection bias.
. There is no description of how applicants were screened for eligibility or how representativeness was considered.
. No justification is provided for the final sample size (N=15), and it is unclear whether this was considered sufficient to detect meaningful changes in pre-post metrics.

**Response #9**: To reduce potential selection bias, the contest was promoted both online and offline through various channels, such as webinars, live media sessions, advertisements, radio and television interviews, flyers, banners, and interviews at Nigeria's university campuses and religious centers. This method aimed to reach a diverse audience across different regions, educational levels, and socio-economic backgrounds, including those without reliable internet or social media access. Although some degree of self-selection is unavoidable in open-call models, this inclusive strategy was intended to expand reach and minimize selection bias. Lines 552-559.

Any English-speaking youth living in Nigeria was eligible for the open call.  The [National Youth Policy (2019)](https://www.google.com/search?sca_esv=3d1e29cf7e253e0e&sxsrf=AE3TifOE3RAgLp3FFlxLe0XJ7RQ5WI4cxw%3A1754075339878&q=National+Youth+Policy+%282019%29&sa=X&ved=2ahUKEwjAw6vsp-qOAxWZX0EAHZigLkkQxccNegQIEBAB&mstk=AUtExfBGzxH53RQuOMrh5cr4WlDilKrZL9TGcaMltKIDkt7-tmravJH0N8RvMflCkAVfB6sb_2tW6ULX0A0P0ofIQ5_eAfLNzLni_vS8auh0bZZaMAd-656cQxOjrctafR7NC9wtRwJEQ2oYfjeN_O1N8J_mRlDam7PfUEjQo3R-WAt5iWJNoFtAeMttwD5uB-A358pf7FrrYP1voHJIuWd-7Pr4r8BpMgx29UfJGbU94DSfVE5Ohkoh5BeGRL2jNP0NDDnA04JGOYuyCWTFb9JhL7jU&csui=3) defines Nigerian youth as individuals aged 15-29 years. Line 119 see citation.

Barriers to implementing the open call included limited understanding of HBV vaccination and limited in-person locations to submit ideas. Specifically, physical responses were only collected at a location in Lagos, which limited participation from individuals in other areas. We have addressed these in the limitations section. Lines 559-560.

The number of finalists was determined pragmatically based on available resources, event logistics, and the capacity to provide meaningful mentorship and support during the bootcamp. The judges selected, to the best of their ability, the most innovative, relevant, feasible, and equitable strategies. Lines 547-550.

Please note that the open call process falls outside the scope of this study and will be detailed in a separate publication. It is briefly introduced here to provide readers with contextual background and insight into the origins of the research.

**Data Collection**
**Comment # 10:** The structure and validation of the pre-post knowledge and research skills survey are unclear. Was the tool piloted? What is the scoring rubric? Were questions validated in a similar setting?

**Response # 10:** Our team has organized over 20 crowdsourcing open calls. Designathon, and bootcamps in Nigeria, other parts of Africa, and the United States. We used the default items that had been developed for prior projects. The survey was then tailored by our Nigerian team to ensure that the HBV-focused components were easily interpretable. Line**s** 307-308.

**Comment # 11:** The 5-point Likert scales and score ranges (e.g., 1 – 45 for knowledge) need explanation. Composite scores should be justified.

**Response # 11:** This has been corrected and explained. Lines 301-306.

**Comment #12:** The qualitative data collection is described in vague terms (“open-ended questionnaires,” “community needs assessment”), with no mention of interview guide development, piloting, or whether data saturation was achieved.

**Response #12:** As explained in Response # 10, our research team has organized several similar projects on diverse topics using these frameworks over the years. We used the default items that had been developed for prior projects and tailored them to the current topic in this manuscript. Saturation was achieved. Lines 312-313.

**Analysis
Comment #13:** Qualitative analysis is said to use Braun & Clarke’s six-step process, but key steps such as theme validation or inter-coder reliability are not described. While the six-step thematic analysis process is referenced, the manuscript does not report whether multiple coders were involved, how disagreements were resolved, or whether any measure of inter-coder reliability was assessed - steps that are critical to ensuring trustworthiness of qualitative findings.

**Response # 13**: In this study, two members of the research team conducted the initial coding, and themes were repeatedly refined through regular discussions and consensus. Although formal inter-coder reliability measures (e.g., Cohen’s kappa) were not calculated, the process involved collaborative checking and resolving discrepancies to improve consistency credibility. Line**s** 328-331.

**Comment # 14**: The PEN-3 model is presented as a post-hoc framework for organizing results, but the rationale for its application during vs. after coding is not explained.

**Response # 14**: The PEN-3 model was used to organize the emerging themes from the thematic analysis to conduct the pilot studies. The PEN-3 model and its rationale are explained in the Methods section. Lines 336-337, 345-349.

**Results
Comment # 15:** Participant Demographics: The sample size is very small (N = 15), heavily skewed by gender (93% female) and age (mean age 25.5). The generalizability of findings is extremely limited and should be acknowledged. The tables are misnumbered – jumping from Table 3 to Table 5.

**Response # 15:** Thank you for your comment. We apologize for the misnumbered tables. We previously explained the reasons for the small sample size in #9. The fact that fourteen out of fifteen participants are female is quite noticeable. However, the authors are not surprised by these results, as the topic of newborn health tends to attract young women. We are encouraged by these women’s enthusiasm for participating in research, especially in a setting where women are often marginalized. Our open call did not discriminate against applicants based on gender.

Regarding the skewed age distribution, most participants were between 20 and 28 years old (N=14). One participant in the team “HEPRECIATE”—a mother-daughter pair, with the daughter as the lead—was 53 years old and was permitted to participate in the bootcamp because the judges found their proposed strategy exceptionally innovative. However, the team eventually left the study because the third (youth) team member was unable to join (reason unknown) and replace the 53-year-old mother for the project. The limited generalizability is acknowledged in the limitations section. Lines 279-285.

**Comment #16:** Quantitative Results:
. The pre-post improvements in knowledge (+21.6%) and skills (+36.4%) are statistically significant, but raw score ranges and IQRs suggest considerable overlap. Although the authors report statistically significant improvements in knowledge and skills, the overlapping interquartile ranges and small sample size suggest that observed differences may be due to variability or chance. A more cautious interpretation is warranted, particularly in the absence of a comparator group.
. No adjustment was made for multiple comparisons, which should be acknowledged.
. Results focus on statistical significance, but clinical or practical relevance is not discussed.

**Response #16:** Thank you very much for this insightful comment. We have carefully revised the results section, particularly the interpretation of the findings, to emphasize a more cautious approach. We also note that the overlapping interquartile range, combined with the relatively small sample size, means that the observed differences should be interpreted with caution and may in part reflect chance variation rather than an actual effect. All these are now reflected in the limitations, including the limitations due to:

•	The lack of a comparator group restricts our ability to attribute improvements solely to the intervention.

•	No adjustment was made for multiple comparisons, which could increase the likelihood of Type I error. Lines 541-546.

**Comment # 17**: Qualitative Findings:
. Themes are clearly organized and relevant, but there is no illustrative data (e.g., participant quotes) to ground them.
. The integration of the PEN-3 model is conceptually sound but would benefit from clearer mapping between themes and PEN-3 components. Figure 3 and Table 5 are helpful, but interpretation is superficial.

**Response # 17:** We agree with the reviewer’s comment to integrate quotes throughout the results and have included illustrative responses and quotes from participants. See table 2 in the manuscript text. Line 339.

The proposed strategies and how they relate to the PEN-3 model have been added in the table S3 in the supplementary file.

**Comment # 18:** Innovation Strategies: Only four out of five teams completed proposals. This missing data point (HEPRECIATE) should be discussed.

**Response # 18**: This has been addressed in the previous response # 15

**Discussion**
The discussion appropriately emphasizes the potential of youth-led, participatory design for improving public health outcomes. However:
**Comment # 19:** There is little reflection on study limitations, including sample size, lack of a control group, non-random selection, and limited external validity.

**Response # 19:** We sincerely appreciate your thoughtful feedback. This study was designed mainly as a descriptive assessment of the bootcamp activities and their role in enhancing research capacity among participants, rather than as a randomized clinical trial or quasi-experimental study. Therefore, its main goal is to offer insights into the feasibility, acceptability, and potential impact of the bootcamp model, rather than to determine causal relationships. In response to your valuable comments, we have expanded the limitations section to clearly state these methodological boundaries. See Limitations starting line 541.

**Comment # 20:** Interpretation of pre-post improvements may be overstated, given the absence of a comparator arm or long-term follow-up.

**Response #20:** The authors acknowledge the absence of long-term follow-up and the absence of a comparator, and have included this limitation in the section. Line 546.

**Comment # 21:** The conclusion hints at broader implications for implementation science, but these are not well-developed. How scalable or sustainable is this bootcamp model? What policy implications arise?

**Response # 21**: As noted in our previous responses, our research team has successfully conducted multiple bootcamps and designathons across diverse themes and locations, demonstrating our ability to implement these interventions at scale. Building on this experience, we are confident that the upcoming clinical trial will provide robust evidence regarding both the scalability and sustainability of the intervention in a broader population. It is important to note that while a policy on HepB-BD vaccination exists in Nigeria, its implementation is often not adequately enforced. Lines 582-585.

**Comment # 22:** There’s no discussion of potential biases (e.g., social desirability, recall) or confounders. Given the non-random selection of participants and reliance on self-reported measures, results may be subject to selection and social desirability biases. In addition, without a control group, improvements in knowledge and skills cannot be causally attributed to the bootcamp alone.

**Response # 22:** We agree with the reviewer that the improvements in knowledge and skills cannot be solely attributed to the bootcamp activities and acknowledge this in the limitations section. Once again, this is a descriptive study and not a randomized clinical trial, so there are no control or comparative groups. Lines 541-546.

**Reviewer #2:**

**Comment # 1:**

General Assessment
This manuscript presents a valuable and innovative approach to engaging youth in Nigeria to develop contextually relevant strategies aimed at improving uptake of the hepatitis B birth dose (HepB-BD) vaccine. The participatory action research framework, supported by the PEN-3 cultural model, provides a strong theoretical foundation. The study is commendable for its focus on community-based intervention, youth empowerment, and cultural responsiveness.

Originality and Relevance
The manuscript introduces an innovation bootcamp model informed by Participatory Action Research (PAR) and Designathon methodologies—rarely described in the Nigerian public health setting and particularly novel in the context of HBV vaccination.

The study is well-timed, addressing a key challenge in global hepatitis elimination efforts—birth dose vaccine uptake in LMICs, where health system barriers and sociocultural factors remain under-addressed. By emphasizing youth leadership, this study adds a new lens to capacity-building frameworks that typically overlook younger demographics.
Strengths
a. Grounded in implementation science and cultural theory.
b. Strong cross-sectoral collaboration between Nigerian and US-based institutions.
c. Relevant to the journal’s mission of advancing global public health equity.
Methodology
The methodology is comprehensive, detailing each phase of the bootcamp from team recruitment, pre-bootcamp preparation, instructional components, and field implementation to evaluation.

Commendable Aspects:
a. Mixed-methods approach enhances data triangulation.
b. Use of the PEN-3 model is contextually apt for Nigeria.
c. Clear and ethical participant recruitment processes.
d. Pre/post knowledge assessments, use of Wilcoxon tests, and thematic qualitative analysis are appropriate.

**Response # 1**: We thank the reviewer for your thoughtful and helpful comments.

Areas for Clarification:
**Comment # 2**: Sample Size Justification: Only 15 participants completed the bootcamp. Please elaborate on how this small sample impacts generalizability or whether this was a pilot by design.
**Response # 2:** The number of finalists was determined pragmatically based on available resources, event logistics, and the capacity to provide meaningful mentorship and support during the bootcamp. The judges selected to the best of their ability the most innovative, relevant, feasible, and equitable strategies. This is reflected in our limitations. Lines 547-550.

**Comment # 3:** AI Screening: The manuscript mentions screening for “>10% AI use” during submission evaluation. This is commendable but would benefit from detail—e.g., what tool was used and the rationale for the cutoff?

**Response # 3**: We used ZeroChartGPT to detect AI-generated content. The tool employs a >10% practical threshold to flag possible AI content. The rationale for the cutoff is to strike a balance between sensitivity and specificity. Like any AI detection tool, it has limitations, including the potential for false negatives where AI-generated text might go undetected. However, we believe the margin of error in this case is minimal. Lines 183-185.

**Comment # 4:** Qualitative Data Saturation: Was thematic saturation reached in the needs assessments?

**Response # 4**: Yes, saturation was achieved during interviews. This information is incorporated in the manuscript. Lines 312-313.

**Comment # 5:** Data Presentation and Analysis
Tables and figures are effectively used. The statistical analyses are appropriate, particularly the use of Wilcoxon signed-rank tests for paired comparisons.
Suggestions:
. Consider visualizing pre- and post-knowledge scores to enhance interpretation.
. Include a schematic or visual summary of the PEN-3 mapping for each proposed strategy.

**Response # 5**: Table 4 shows the pre- and post-knowledge and research skills scores. Line 392.

The PEN-3 domains in relation to the strategies have been added in the supplementary file. Table S3.

**Comment # 6:** Ethical Considerations
The manuscript demonstrates adherence to ethical standards:
Ethical approval and informed consent were obtained.
Participant support (e.g., travel, meals) was provided.
Clarifications Needed:
. Confirm whether pilot proposals underwent independent IRB review.
. Address any risk of dual publication: the manuscript cites prior 4YBY bootcamps funded by NIH. Are any datasets or textual components overlapping?

**Response # 6:** The pilot studies have received ethical approval. Line 296.

There are no concerns regarding duplicate publication. While the 4YBY project also addresses HIV testing through similar frameworks (Designathon and YPAR), this study is distinct in scope and focus. There is no overlap or duplication of data between the two projects.

**Comment # 7:** Interpretation and Discussion
The discussion is thoughtful and well-grounded in theory and practice. The authors compare their findings to relevant global health and implementation science frameworks and acknowledge both strengths and limitations.
Suggestions:
. The gender balance was highly skewed (93.3% female). Could this have influenced strategy design, especially in patriarchal settings where male decision-makers may dominate?
. Further discussion on how the model could be institutionalized (e.g., through government health departments or university curricula) would strengthen the policy implications.

**Response # 7:** The proportion of female participants—fourteen out of fifteen—is noticeable. However, this outcome is not unexpected, as issues related to newborn health are often of particular interest to young women. We are encouraged by the enthusiasm and commitment demonstrated by these young women, especially in a context where women are frequently marginalized in research and decision-making. It is important to note that our open call did not in any way restrict or discriminate against submissions based on gender.

Nigeria has policies in place for hepatitis B vaccination at birth. The findings of this study could strengthen policy implementation by ensuring strategies are culturally informed, tailored to community needs, and built on existing community assets and competencies, factors essential for community empowerment and sustainable interventions**.** Lines 572, 582-585.

**Comment #8: Conclusion**
The manuscript ends with a strong, practical call to action. The authors provide clear next steps and a justification for further investment in youth-led capacity building for immunization and public health innovation.
. Language & Editing: The text is clear and well-written; minor grammatical improvements may be needed, please proof read the manuscript.
. Supplemental Files: Ensure all referenced figures/tables (e.g., S1–S4) are uploaded and correctly formatted.

**Response # 8:** The authors have proofread for grammatical accuracy of the manuscript and adjusted the supplementary files format. Thank you for pointing this out.

**Reviewer # 3**

**Comment # 1:** The title does not accurately describe what is discussed throughout the manuscript. The title references improved training on hepatitis B research, but the manuscript is focused on the implementation of a bootcamp. Consider revising the title to better match the contents of the manuscript.

**Response # 1:** Thank you for your comment on the title. The bootcamp represents an innovative, hands-on training program designed to equip emerging researchers with practical skills and knowledge on hepatitis B and its prevention. Lines 146-148.

**Comment # 2**: Abstract is 320 words; it must not exceed 300.

**Response # 2:** In response to your comment, we carefully proofread the abstract and confirmed the wordcount being 298 words.

**Comment # 3:** Line 61: The abstract mentions a hepatitis B birth dose vaccination coverage of 30% in Nigeria, which is incorrect. According to the WUENIC data, Hepatitis B birth dose vaccination coverage within 24 hours of birth in Nigeria was 52% in 2023 and has been consistent since 2019. The reference included in the manuscript also shows vaccination coverage at 52% from 2019 – 2021. Please review the included citation (#7) as well as WHO WUENIC data on Hepatitis B birth dose vaccination coverage in Nigeria and correct this statement.

**Response # 3:** We thank you for this useful insight. The authors completely agree with this observation and have updated the indicators with adequate referencing to WHO/UNICEF Estimates of National Immunization Coverage (WUENIC) 2024. Line 64, 108.

**Comment # 4:** Line 78-81: conclusion should speak directly to study aim. If implementation of developed strategies were not complete in time for this manuscript, then the study aim should be revised to speak to what this manuscript is describing.

**Response # 4:** The implementation strategies were indeed developed during the bootcamp. However, piloting these strategies is beyond the scope of this manuscript. In response to the comment regarding lines 78–81, we have revised the conclusion to align more directly with the study aim, emphasizing the development of strategies rather than their implementation, which was not commenced at the time, for inclusion in this paper. Lines 561-565.

**Comment # 5:** Line 104: include year of HBV prevalence referenced here.

**Response # 5:** The prevalence year has been included. Lines 92-93.

**Comment # 6:** Line 107-109: “HBV vaccine initiated within 24 hours of birth, followed by three additional doses (typically at 6, 10, and 14 weeks after birth), provides durable immunity, substantially decreasing the risk of HBV and the incidence of liver cancer in nearly 100% of cases [5].” The proportion of ‘nearly 100% of cases’ does not seem accurate and is not seen in the included citation. Would suggest reviewing the 2017 WHO position paper on Hepatitis B vaccines and possibly referencing that document instead of the reference from HHS. Please confirm proportion and correct based on updated reference.

**Response # 6:** All hepatitis B vaccines are highly effective in preventing HBV infection. Overall, hepatitis B vaccination produces sero-protection in 98% of healthy term infants. Vaccine response among infants does not vary considerably by maternal HBsAg status or HBIG administration. As a postexposure immunoprophylaxis measure for infants born to an HBsAg-positive birth parent, hepatitis B vaccine without HBIG is 75% effective at preventing perinatal HBV transmission, but when combined with HBIG, the effectiveness is 94%. Ref. [5]. Lines 97-103.

**Comment # 7**: Line 109-111: Suggest including timeframe for this statement since the number of countries with HepB-BD in their immunization schedule has increased from 14 and will continue to increase given Gavi’s support of HepB-BD introduction.

**Response # 7**: This has been updated. Lines 103-105.

**Comment # 8:** Line 113-114: see comment in abstract section about this statistic. It is currently incorrect and needs to be revised.

**Response # 8**: The authors appreciate the reviewer’s attention to details. We believe the reviewer is referring to the vaccine coverage, that will be line 111. This has been revised in the introduction section, as the abstract ended on line 78 previously. Lines 107-108.

**Comment # 9:** Line 114-115: “The failure to engage end-users in planning and implementation is a major driver of unsuccessful interventions addressing low vaccination coverage [5].” In the case of HepB-BD, aren’t the end-users newborns? Not sure if this statement makes sense in this context, consider revising.

**Response # 9:** In the context of our study end-users are mothers of the newborns as they are the primary caregivers and responsible for their newborn health. Line 108.

**Comment # 10:** Line 116: Not familiar with terms “Global South” and Global North”, please define what countries are being referred to here.

**Response # 10**: The terms Global South and Global North are commonly used to describe socio-economic and political differences in the world, not just geographical differences. Global South refers to low- and middle-income countries (e.g., in Africa, Latin America, Asia, and parts of Oceania). Global North refers to economically developed, industrialized, and historically dominant high-income nations (e.g., in Europe, North America, and parts of East Asia and Oceania). Lines 111-112.

**Comment # 11:** Line 118-119: “Many projects in the global South have failed or faced problems because the primary beneficiaries were not involved in initiating, designing, and delivering the project [8]”. Again, in the case of HepB-BD, the primary beneficiaries (e.g., newborns) aren’t able to be involved in initiating, designing, or delivering the project. This statement does not make sense in this context, consider revising or removing.

**Response #11:** In the context of our study end-users are mothers of the newborns as they are the primary caregivers and responsible for their newborn health. Line 108.

The bootcamp participants are involving the end-users in the pre-implementation phase of their project by conducting SWOT analysis and DCE. However, the pilot studies are beyond the scope of this study.

**Comment #12:** Line 126: “young people represent a significant portion of the population; 45.46% are between 15 and 35 years old”. Please change ‘are’ to ‘were’ since referencing a proportion from 2021.

**Response # 12:** This comment is duly noted, and the sentence corrected. Line 123.

**Comment # 13**: The connection between young people and how they can engage the community or drive community change needs to be emphasized. Line 121-122 speaks to community ownership overall but the connection with youth needs to be strengthen in the introduction to rationalize why they are the best target audience for this study.

**Response # 13**: The connection between young people and how they can engage the community or drive community change needs has been emphasized in Line 120-127.

**Comment # 14**: Line 145-146: what is hepatitis B research training? This term is vague and does not clearly convey what is being advanced. Is it training others on how to do hepatitis B research or training others on hepatitis B burden and disease? Please clarify term.

**Response # 14:** This has been clarified in Lines 146-148.

**Comment # 15:** Line 146: “By outlining our journey from generating ideas to conducting pilot fieldwork…” the outline for conducting pilot fieldwork is missing in this manuscript. Since the bootcamp was conducted a year ago (June-July 2024) perhaps there is more substantial data available from pilots that can be included in this manuscript write up. Please include an outline and description of pilot projects for context.

**Response # 15**: The pilot studies are currently being implemented and beyond the scope of this paper. The bootcamp ended with the generation of implementation strategies. Lines 152-154.

**Comment # 16:** Line 152: Was the goal focused on overall HepB-BD vaccination or timely (within 24 hours)?

**Response # 16**: The focus of the project is on hepatitis B birth dose vaccination. Birth dose is defined by WHO as a dose of monovalent vaccine delivered within the first 24 hours after birth. Lines 149-151.

**Comment # 17**: It is unclear if there was any specific training/education/sensitization included in the bootcamp to educate youth on hepatitis B burden, disease, and prevention. This detail needs to be explained in the methods section. Were youth expected to know anything about hepatitis B before being tasked with generating innovative ways to improve HepB-BD uptake?

**Response #17:** The uniqueness of crowdsourcing lies in attracting applicants with a basic knowledge of the research area, such as infectious diseases, particularly hepatitis B in this case, to respond to the open call and submit a coherent write-up on the topic. Despite this, knowledge of the research area was incorporated in the training schedule is included in the manuscript. See Table 1: Setting the stage includes educating participants about hepatitis B. Line 256.

**Comment # 18**: Line 166-169: Run-on sentence. Please rephrase for clarity.

**Response #18:** This is noted and has been improved. The sentence seemed run-on because of the journal instructions that required the authors to provide a detailed explanation of the ethical approval. Lines168-171.

**Comment # 19**: Line 172-173: “The research team received 349/396 submissions electronically (WhatsApp, email, and the website) and 11/396 in person”. The numbers 349 + 11 add up to 360 not 396. Where did the other 36 entries come from? Also, what is meant by “the website”?

**Response #19**: We appreciate the reviewer’s feedback. This has been corrected. Line182-183.

**Comment # 20:** Line 174-175: Include a description of how all entries were scanned for AI usage. What was used for this scan? Include citation of tool, if available.

**Response # 20:** We used ZeroChatGPT to detect AI-generated content. The tool employs a >10% practical threshold to flag possible AI content. The rationale for the cutoff is to strike a balance between sensitivity and specificity. Like any AI detection tool, it has limitations, including the potential for false negatives where AI-generated text might go undetected. However, we believe the margin of error in this case is minimal. Lines 184-186.

**Comment # 21**: Line 177 implies that all entries were submitted by a “team”. Was this part of the submission requirements? Were youth asked to submit as a team vs an individual? This needs to be explained more clearly in the methods.

**Response #21**: We appreciate your insightful comment. The applicants were instructed to submit proposed strategies as a team consisting of 2-5 persons. This has been added in the method section. Lines 181, 208.

**Comment # 22**: Line 179-181 implies that finalists were tasked with designing and refining their strategies prior to the bootcamp, where all the research training took place. Were they provided feedback on their idea or given any guidance during the Designathon? The order of operations for this study is unclear, please clarify.

**Response # 22:** The participants received mentorship on refining their strategies during the Designathon, which was held before the bootcamp. Lines 188-194.

**Comment # 23:** Line 180-181: finalists were asked to refine strategies to enhance HepB-BD uptake and awareness within Nigerian communities. Were communities specified in any way or decided upon based on team? Could imagine that communities across Nigeria vary greatly and would require different approaches. Since teams were all from Southern Nigeria, did their interventions only focus on these communities? Southern Nigeria has higher rates of HepB-BD vaccination uptake compared to Northern Nigeria (according to 2021 MICS/NICS report), this is an important point that should be mentioned in the discussion of the paper, perhaps as a limitation given the lack of attention provided to communities in Northern Nigeria, where the burden of hepatitis B is greater.

**Response #23:** The finalists were expected to implement their strategies within their own communities, where their familiarity with local contexts facilitated their acceptance as researchers, particularly given the cultural specificities in Nigeria. The authors acknowledge that as a limitation, as no participants originated from the Northern region of the country. However, this was beyond our control, as the open call was accessible to all Nigerian youth, and participation was determined by applicant submissions. Lines 550-560.

**Comment # 24:** Line 191: “The five teams that made it past the Designathon…” this phrase needs to be better explained. Did the other 5 teams drop out? Were they lost to follow up?

**Response # 24**: The judges evaluated the strategies of 10 teams at the end of the Designathon, and the top five that emerged were invited to the bootcamp. Lines192-194

**Comment # 25:** Line 196-198: Were team facilitators knowledgeable about hepatitis B at all? Were they provided any training or education on the disease burden or overall prevention strategy? If so, this needs to be explained in the methods section.

**Response #25:** Lines 209-212 list the facilitators from various fields, including public health, indicating that they are experts in the primary prevention of HBV.

**Comment # 26**: Line 232: “The judges comprised community leaders…”, should read “The judges comprised ‘of’ community leaders”.

**Response # 26**: Noted and corrected. Thank you for pointing it out. Line 250.

**Comment # 27:** Line 248: It is unclear who the ‘target population’ is. Does it vary by team? Is it mothers, caregivers, newborns, community leaders, traditional birth attendants?

**Response # 27:** Each team had a different approach to addressing the low vaccine uptake. Therefore, they targeted various populations within their communities. Please refer to supplementary file table S3.

**Comment # 28:** Line 259: Placement needs to be better explained in this paragraph. Was it that teams were judged and if received first place they were given seed funding to pilot their proposed strategies? How does this fit with the timeline presented in Table 1?

**Response # 28:** All five teams received seed money from the project to commence their various pilot study (screening, enrollment of participants, engaging with community leaders, and other related activities). The amount of the prize money differed by the winning position of each team. In Table 1, this corresponds to the last day of the bootcamp immediately after the pitch. This has been added to the table and in Line 275.

**Comment # 29:** Line 271-272: A virtual refresher course was completed months after the bootcamp ended. Please describe the expected timeline of implementing the pilot. If selected for first place for example, what is the expected timeline for the pilot? Is it after the refresher course is completed?

**Response # 29:** The refresher course was held several weeks after the bootcamp to prepare for the start of the pilot studies. Teams were ready to conduct the pilot studies at different times due to logistical issues and the specifics of local settings (e.g., many participants are students with school commitments, engaging community leaders required long wait times to schedule appointments, obtaining permission from local health authorities to recruit participants, and other challenges). However, this is beyond the scope of our study, which focuses on the bootcamp implementation.

**Comment # 30:** Line 291: indicate at what p-value was an estimate considered statistically significant.

**Response # 30:** Thank you for this suggestion. This section has been revised to indicate at what p-value the estimate was considered significant. *“The Wilcoxon Signed-Rank Test (Exact) was used to compare pre- and post-training data, and results were reported as mean and median differences at 5% level of significance.”* Line 321, 322.

**Comment # 31:** Line 318: the term ‘existential’ should be defined in line 310 where it is first mentioned rather than in line 318.

**Response # 31**: This is noted and corrected. Line 350.

**Comment # 32:** Line 328 mentions that 5 teams completed the bootcamp but in line 261 it mentions that all but one team submitted study proposals that were approved by the research team.

**Response # 32**: This is noted and corrected. Four out of the five teams that participated in the boot camp submitted their study proposals. Lines 204, 279, 280.

**Comment # 33:** The study emphasizes the focus on youth; however, the mean age of participants is 25.5 years (ranging from 20 – 53 years old). Given this age, participants were all adults, rather than youths. This is an important clarification that changes the overall assertion of the manuscript and needs to be addressed in the discussion.

**Response # 33:** The National Youth Policy defines Nigerian youth between the ages of 15 and 29. All participants but one were 20-28 years. Line 119**.**

**Comment # 34**: Line 339: Table 2 includes min/max but the text references IQR. Would suggest being consistent on which one is reported. If using IQR, be sure to spell out acronym at first use.

**Response # 34**: Table 2 (now Table 3) reports socio-demographic statistics of bootcamp participants. Here we reported the mean and median (min; max) of participants’ age in the table and reported the mean in the text. However, in Table 4 (formally Table 3), we reported the mean, median, and interquartile range for knowledge and research skills pre- and post-training. To spell this put, we have clarified and mentioned the interquartile range as a note. Lines 382-385.

**Comment # 35:** Line 340: were the difference in pre-test scores described here significant based on the exact test performed? This needs to be stated and p-value included, if yes.

**Response # 35:** Thanks for bringing this up. We have shifted the p-values next to the mentions of the median differences upfront to indicate the statistically significant differences between pre- and post-scores for both knowledge and research skills. Lines 389-390.

**Comment # 36: Table 3:** In the post column, there is a maximum research skills score of 101. Is it possible to score over 100? Are these numbers and proportions correct? This needs to be clarified in the table. Please be sure to be consistent with the number of places after the decimal point for all proportions presented in Tables.

**Response # 36:** These are not percentages but numbers indicating the sum of scores on the Likert scale. Lines 303-307.

**Comment # 37:** Line 351-354: Are these open-ended questions or themes? If not, then %’s should also be included to have a sense of how many participants mentioned the same topic.

**Response # 37:** These are the key themes that emerged from the thematic analysis of the survey. The paragraph has been rephrased to the actual questions. Lines 310-312.

**Comment # 38:** Line 375: Again, how was youth defined in this manuscript? Based on the age of participants presented in Table 2, individuals are technically adults, not youth.

**Response # 38:** The National Youth Policy defines Nigerian youth between the ages of 15 and 29. All participants but one were 20-28 years. Line 119.

**Comment # 39:** Table 5: Under the perceptions domain, TBAs are listed under existential but “believe in traditional/alternative medicine” is listed under negative. This seems discordant, please address/clarify in text if this is indeed the correct placement for each. Table 5: This table would be easier to read if each new line was denoted with a bullet point

**Response # 39**: TBAs and traditional medicine are two distinct concepts. TBAs conduct antenatal clinics and assist with deliveries much like midwives. The main differences lie in the setting and environment, such as home-based care, close relationships with clients, the use of local languages, and other factors that promote clients’ comfort and trust. TBAs are integral to the community since they are a part of its fabric, and whether their practices are beneficial or not, they cannot be separated from the community. Belief in traditional or alternative medicine also exists within the African community and offers certain benefits. However, in the context of vaccination, it acts as a negative influence that hinders the prevention of vaccine-preventable diseases, which traditional medicine cannot achieve. Bullet points have been added in Table 5. Line 437.

**Comment # 40:** Line 397: Again, not sure authors can make the claim that this bootcamp was youth led given the ages of the participants. Either define the accepted age range for youth in the introduction of this manuscript or rephrase/remove the term youth as a descriptor for participants.

**Response # 40:** This has been addressed in responses above #38.

**Comment # 41:** Line 404: Again, the notion of ‘involving the audience in design’ is not applicable here as the target audience for HepB-BD uptake is newborns. If the target audience is mothers/caregivers then the argument of involving the audience works –although it would mean that teams were led by reproductive-aged participants rather than youths. Please make sure it is clear through the manuscript who the target audience/primary beneficiaries are.

**Response # 41:** The target audience for the Innovation Bootcamp is the Youth. Lines 119-121/In the context of our study the end-users of the interventions developed by the Youth participating in the bootcamp are mothers of the newborns as they are the primary caregivers and responsible for their newborn health. Line 108.

**Comment # 42:** Line 442: Is there a reference that can be included for the “Ayanfe” chatbot referenced here? Was this chatbot created by the team for the purpose of this project or did it already exist? This needs to be clearly explained in the manuscript. Line 462: Is the chatbox referenced here the same chatbox mentioned in line 442? If not, include name of the chatbox and citation if available.

**Response # 42**: The chatbox “Ayanfe” was created by one of the teams as an implementation strategy specifically for this project to improve Hepatitis B Birth Dose vaccinations. See clarification on Lines 482-483.

**Comment # 43:** The discussion describes what was planned and proposed but does not speak to what was implemented at all. This was part of the study aim and described in the methods but is missing from the results and discussion sections of the paper. Please include.

**Response # 43**: The aim of this study was to describe an innovation bootcamp and its impact on building research capacity. Lines 149-151. The implementation of the bootcamp is the training of bootcamp participants. The implementation of the proposed strategies is outside the scope of this paper.

**Comment # 44:** Line 473-474: Again, the claim of youth-driven research needs to be modified given the ages of participants. Also, a reference is needed for the statement “Nigeria has a large youth population,” be sure to include the age range for the ‘large youth population’ referenced.

**Response # 44**: The National Youth Policy defines Nigerian youth between the ages of 15 and 29. All participants but one were 20-28 years. Line 119.

**Comment # 45:** Line 477: Include citation for Ayange Chatbot if available.

**Response # 45:** This proposed strategy by a team is still in the early stages. Citation is not available. Lines 482-483.

**Comment # 46:** Line 493: Did the bootcamp address any of the mentioned challenges? Were these challenges faced by implementing teams or just stated from previous literature? Were teams expected to design intervention with common challenges in mind? Please clarify in the text.

**Response # 46**: The expected challenges were drawn from existing literature, including publications of our research team’s projects in LMICs on an array of topics, e.g., HIV, HPV, HT, and discussed during the bootcamp. Lines 538-540: “real-world application, preparing participants to work closely with communities in designing and implementing public health interventions”. Teams were also expected to design their interventions with common challenges in mind.

**Comment # 47:** Line 502: The “execution of intervention strategies” were not clearly described in this manuscript. Please include.

**Response # 47:** This has been corrected. Line 563.

**Comment # 48:** Line 516-517: This is the one sentence in the manuscript that mentions that piloting of strategies is occurring. This should be described in more detail in the methods and results sections, describing the expected timeline, location of implementation, and any other relevant details.

**Response # 48:** The pilot studies are beyond the scope of this paper. However, the authors included the estimated timeline of six months for the pilot studies that are conducted in the participants’ respective communities. Line 581-582.
